# Ferroptosis Induction in Pentylenetetrazole Kindling and Pilocarpine-Induced Epileptic Seizures in Mice

**DOI:** 10.3389/fnins.2019.00721

**Published:** 2019-07-17

**Authors:** Xiao-Yuan Mao, Hong-Hao Zhou, Wei-Lin Jin

**Affiliations:** ^1^Department of Clinical Pharmacology, Xiangya Hospital, Central South University, Changsha China; ^2^Hunan Key Laboratory of Pharmacogenetics, Institute of Clinical Pharmacology, Central South University, Changsha, China; ^3^Engineering Research Center of Applied Technology of Pharmacogenomics, Ministry of Education, Changsha, China; ^4^National Clinical Research Center for Geriatric Disorders, Changsha, China; ^5^Centers for Translational Medicine, Ruikang Hospital, Guangxi University of Chinese Medicine, Nanning, China; ^6^Key Laboratory for Thin Film and Microfabrication Technology of Ministry of Education, Department of Instrument Science and Engineering, School of Electronic Information and Electronic Engineering, Institute of Nano Biomedicine and Engineering, Shanghai Jiao Tong University, Shanghai, China

**Keywords:** epilepsy, seizure, ferroptosis, lipid peroxidation, pentylenetetrazole, pilocarpine

## Abstract

Epilepsy is a serious neurological disorder and is characterized by recurrent and unprovoked seizures. A critical pathological factor in the seizure genesis is neuronal loss. Until now, apart from the known regulatory cell death pathways, ferroptosis is a newly discovered type of cell death with the features of iron accumulation and the excessive production of lipid reactive oxygen species (ROS). In our present work, it was illustrated that ferroptosis occurs in murine models of pentylenetetrazole (PTZ) kindling and pilocarpine (Pilo)-induced seizures. In both of these seizure models, treatment with ferroptosis inhibitor ferrostatin-1 (Fer-1) efficiently alleviates seizures. This was achieved through elevated levels of glutathione peroxidase 4 (GPX4) and glutathione (GSH) as well as inhibitions of lipid degradation products including 4-hydroxynonenal (4-HNE) and malonaldehyde (MDA), iron accumulation, and PTGS2 mRNA in the hippocampus. It was concluded that ferroptosis is involved in seizure genesis in PTZ- and Pilo-treated mice, while the suppression of ferroptosis mitigates PTZ kindling, and Pilo-induced seizures in mice.

## Introduction

Epilepsy is one of the most prevalent and severe chronic neurological disorders, afflicting approximately 50 million people worldwide. It often features the generation of spontaneous and recurrent seizures in the affected brain area including the hippocampus (Fisher et al., 2014; Mao et al., 2014; Li et al., 2019), finally disrupting proper brain function. Nowadays, more than 20 anti-seizure drugs (ASDs) have been approved for the treatment of epilepsy. Nevertheless, nearly 30% of patients fail to achieve seizure control (Kwan et al., 2011; Fiest et al., 2017). Additionally, the traditional ASDs show various and serious adverse reactions as a result of their action on ubiquitously distributed targets that are involved in physical processes (Sarma et al., 2016). This has spurred the identification of alternative targets to develop satisfactory anti-seizure therapies.

Ferroptosis, a recently discovered regulated cell death (RCD) which can be manipulated pharmacologically and genetically and under the control of intrinsic molecular mechanism (Stockwell et al., 2017), is characterized with iron-dependent lipid peroxidation (Dixon et al., 2012; Li et al., 2018c; Mao et al., 2018). It is distinct from other cell death modalities including apoptosis, necroptosis and autophagy, at morphological, biochemical, and genetic levels. Specifically, mitochondrial shrinkage and a condensed outer membrane are the features of ferroptosis (Dixon et al., 2012). It has been extensively reported that ferroptosis is involved in the etiology of diverse neurological disorders such as Alzheimer’s disease, Parkinson’s disease, stroke, and traumatic brain injury (Do Van et al., 2016; Tuo et al., 2017; Wenzel et al., 2017; Zhang et al., 2018). However, the role of the ferroptosis process in the seizure genesis remains unclear, especially in pentylenetetrazole (PTZ) kindling and pilocarpine (Pilo)-induced seizures. In our present study, we provided the direct evidence for the occurrence of ferroptosis in murine models of PTZ kindling and Pilo-induced seizures. We found that treatment with ferroptosis inhibitor ferrostatin-1 (Fer-1) potently alleviated seizure severity and frequency.

## Materials and Methods

### Animals and Ethics Statement

Male C57BL/6J mice (8–10 weeks old) were provided from the Animal Centre of Central South University and maintained in a constant environment (24 ± 2°C, 12 h light/12 h dark, 50–70% humidity) with *ad libitum* access to standard food and water. All animal care and procedures throughout the study were approved by the Ethical Committee of the Animal Centre of Central South University.

### Establishments of PTZ Kindling and Pilo-Induced Seizure Models

#### PTZ Kindling

The C57BL/6J mice were intraperitoneally injected with PTZ (35 mg/kg, Sigma-Aldrich, United States) once every other day for eleven injections (Mizoguchi et al., 2011) and mice exhibiting more than three consecutive stage 4 seizures were considered to be kindled. Behavioral seizures were analyzed for the subsequent 90 min after the last injection of PTZ according to a modified Racing scale (Kay et al., 2015; Matsuda et al., 2015): stage 0, no response; stage 1, immobility; stage 2, rigidity; stage 3, head bobbing and circling; stage 4, intermittent rearing and falling; stage 5, continuous rearing and falling; and stage 6, tonic-clonic convulsions and rapid jumping. Animals that died were assigned stage 6 during the experiments. Ferrostatin-1 (Fer-1, Selleck, United States) were administered intraperitoneally at the 8th day after PTZ injection with a dose of 2.5 μmol/kg for 2 consecutive weeks. The dosage of Fer-1 was selected according to a previous report (Wang H. et al., 2017).

#### Pilo Model

The ramping-up dosing protocol was selected for the preparation of the Pilo-induced seizure model (Muller et al., 2009). In brief, the C57BL/6J mice underwent repeated low-dose treatment by intraperitoneal application of Pilo (100 mg/kg, Sigma-Aldrich, United States) every 20 min until onset of limbic seizure. Usually, three injections are sufficient for the induction of continuous seizure activity. Methylscopolamine (1 mg/kg, Sigma-Aldrich, United States) was injected intraperitoneally 30 min prior to Pilo in order to evade peripheral cholinergic side effects. And after 90 min of continuous limbic seizures, mice received diazepam (10 mg/kg, XiangYa Hospital, China) for the termination of seizures. The control group was injected with methylscopolamine and diazepam, like the Pilo-treated mice, except for three injections of saline instead of pilocarpine. Fer-1 was pretreated with a dose of 2.5 μmol/kg for 2 consecutive weeks before Pilo injection.

### Transmission Electron Microscope (TEM)

Mice from different groups were deeply anesthetized with 10% (g/ml) chloral hydrate and transcardially perfused with 0.1 M phosphate buffer saline (PBS, pH = 7.4), followed by the fixations of 4% paraformaldehyde (PFA), and 2% glutaraldehyde. The tissues were cut into 100 nm-thick sections and then were stained with uranyl acetate and lead citrate. Morphological mitochondrial features were observed under a JEM2000EX transmission electron microscope (TEM; JEOL, Tokyo, Japan).

### Western Blot Assay

Western blotting was conducted according to our previous descriptions (Li et al., 2018b). Briefly, hippocampal tissues were lysed in high KCl lysis buffer containing 10 mM Tris–HCl, pH 8.0, 140 mM NaCl, 300 mM KCl, 1 mM EDTA, 0.5% Triton X-100, and 0.5% sodium deoxycholate with 1 mM phenylmethylsulfonyl fluoride (Roche, United Kingdom). The supernatant was quantified using a commercial BCA kit (Beyotime Biotechnology Institute, China). Protein samples were separated by SDS-polyacrylamide gels (SDS-PAGE) and transferred electrophoretically to polyvinylidene fluoride membranes. After blocking, the membranes were incubated with 4-hydroxynonenal (4-HNE) (Mouse, 39–122 KDa, MAB3249, 1 μg/ml, Novus, United Kingdom), GPX4 (Rabbit, 22 kDa, ab125066, 1:5000, Abcam, United Kingdom) and β-actin (Mouse, 43 kDa, A5441, 1:10000, Sigma-Aldrich, United States) overnight. The next day, after washing, the membranes were then incubated with secondary IgG goat anti-rabbit (A9169, 1:10000, Sigma-Aldrich, United States) or rabbit anti-mouse antibody (A9044, 1:10000, Sigma-Aldrich, United States). Immunodetection was performed using an enhanced chemiluminescence kit and the intensity of protein bands was analyzed by Quantity One software (BioRad, United States).

### Real-Time Quantitative PCR

After drug treatment, total RNA from tissues or cell cultures was obtained using TRIzol reagent (Invitrogen, United States) following the manufacturer’s protocols. Then, 1 μg total RNA for each sample was reverse transcribed into complement DNA using the SYBR Green RT Kit (Takara, Japan). Real-time PCR was carried out using SYBR Green PCR Master Mix (Takara, Japan). Samples were analyzed on a LightCycler Roche 480 qPCR instrument with absolute quantification settings. PCR conditions were displayed as follows: 30 s hot start at 95°C followed by 40 cycles of 5 s at 95°C, 30 s at 55°C, and 30 s at 72°C; 30 s melting curve at 95°C. All samples were determined in triplicate, and differences in mRNA levels were calculated using the δδCt method, with β-actin as an internal reference control. The following primer sequences were used: PTGS2: Forward: 5′-GGGAGTCTGGAACATTGTGAA-3′ and Reverse: 5′-GTGCACATTGTAAGTAGGTGGACT-3′. β-actin: Forward: 5′-GTGACGTTGA-CATCCGTAAAGA-3′ and Reverse: 5′-GCCGGACTCATCGTACTCC-3′.

### Measurements of Malonaldehyde (MDA) and Glutathione (GSH) Levels

Measurements of GSH and MDA levels were detected using their corresponding commercial kits (S0055 for GSH and S0131 for MDA, Beyotime Technology Institute, China) according to manufacturer’s instructions.

### Detection of Iron Content

The iron concentration from each group was determined using an Iron Assay Kit (ab83366, Abcam, United Kingdom) following the manufacturer’s protocols.

### Data Analysis

Experimental data was presented as mean ± SD and Prism 5.0 software (GraphPad Software, La Jolla, CA, United States) was used for analysis. A statistical significance was identified using Student’s *t* tests between two groups and between three or more groups by analysis of variance, using One-Way ANOVA with the Bonferroni test. Statistical differences with *p* values less than 0.05 were deemed significant.

## Results

### Occurrence of Ferroptosis in Murine Models of PTZ Kindling and Pilo-Induced Seizures

**FIGURE 1:**
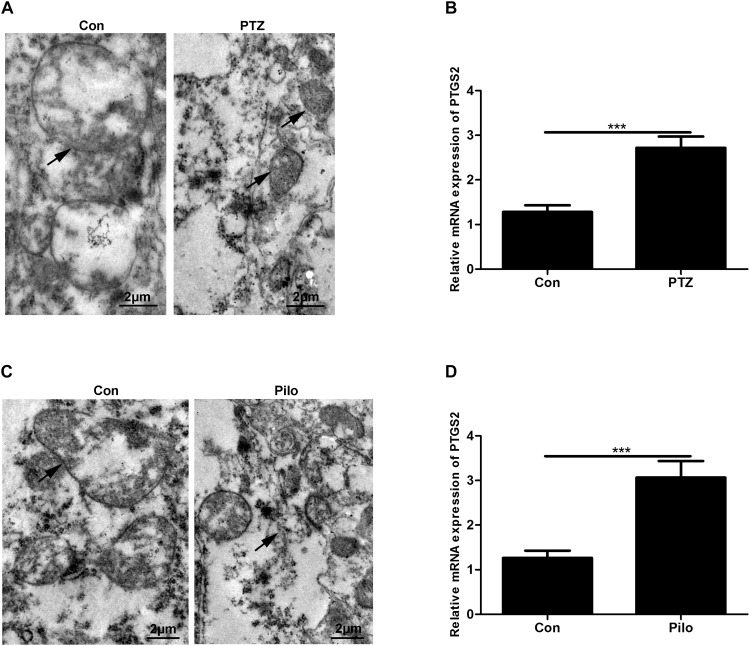
Ferroptosis occurs in PTZ kindling and Pilo-induced seizures in mice. **(A**,**C)** Ultrastructure of neurons in PTZ- and Pilo-treated mice. Arrows indicate the representative mitochondria. **(B**,**D)** Quantitative analysis of PTGS2 mRNA in the hippocampus of PTZ- and Pilo-treated mice (^∗∗∗^*p* < 0.001, *n* = 6).

First, we explored whether ferroptosis occurred in PTZ kindling and Pilo-treated mice through morphological observation of mitochondria using TEM and the detection of PTGS2 mRNA, a previously identified ferroptotic marker, using real-time quantitative PCR (Jiang et al., 2015). Our results revealed that smaller mitochondria and the upregulation of PTGS2 mRNA were found in murine models of PTZ kindling or Pilo-triggered seizures (Figure 1), indicating the presence of ferroptosis in these two models.

### Fer-1 Attenuates Seizures in PTZ Kindling and Pilo-Treated Mice

**FIGURE 2:**
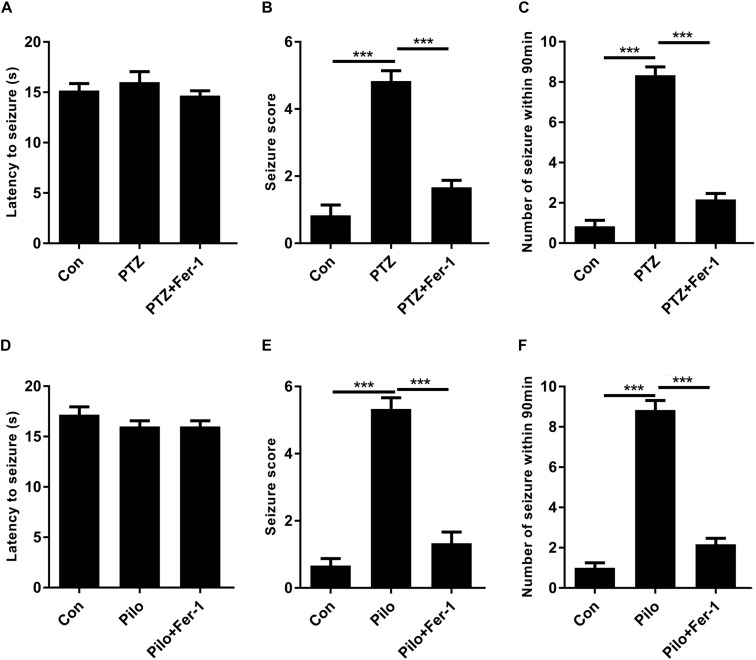
Fer-1 alleviates PTZ kindling or Pilo-induced seizures in mice. **(A–C)** Evaluations of latency to seizures, seizure score and number of seizures within 90 min in PTZ-treated mice after Fer-1 treatment. **(D–F)** Evaluations of latency to seizures, seizure score, and number of seizures within 90 min in Pilo-treated mice after Fer-1 treatment (^∗∗∗^*p* < 0.001, *n* = 9).

PTZ- and Pilo-treated mice both exhibited at least stage 5 seizures while mice in the control group did not show any signs of seizure (Figure 2). We also observed an average of eight and nine seizures in PTZ- and Pilo-treated mice within 90 min of behavioral observation (Figure 2). However, treatment with Fer-1, a specific ferroptosis inhibitor (Dixon et al., 2012; Shintoku et al., 2017), significantly decreased seizure severity (decreased seizure score) and frequency (decreased number of seizures within 90 min) in murine models of PTZ kindling as well as Pilo-induced seizures. No significant difference was found among the three groups in terms of the latency to seizures.

### Fer-1 Decreases Iron Accumulation and PTGS2 mRNA in the Mice Hippocampus of PTZ Kindling and Pilo-Induced Seizures

**FIGURE 3:**
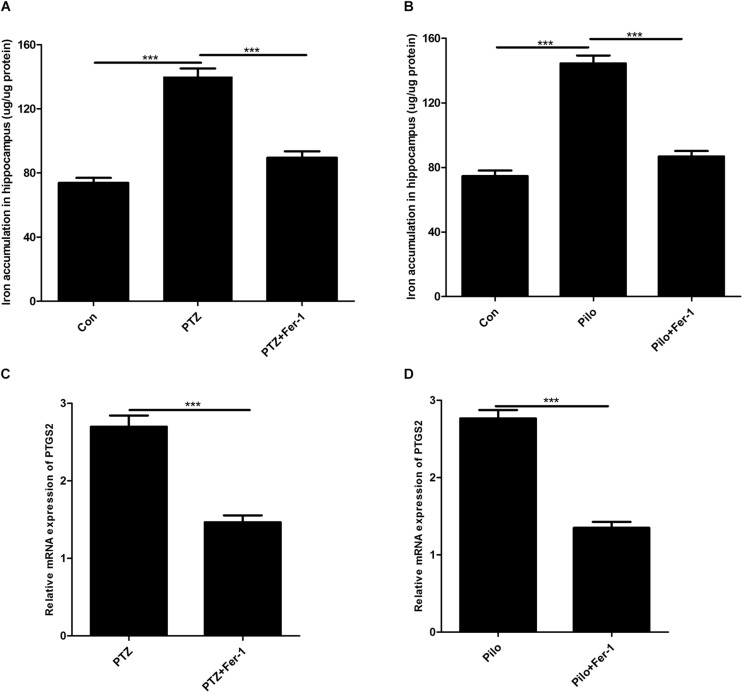
Fer-1 decreases iron accumulation and PTGS2 mRNA in the hippocampus of PTZ- and Pilo-treated mice. **(A**,**B)** Quantitative analysis of iron content in the hippocampus of PTZ- and Pilo-treated mice (^∗∗∗^*p* < 0.001, *n* = 6). **(C**,**D)** Effect of PTGS2 mRNA in the hippocampus of PTZ kindling and Pilo-induced seizure models after Fer-1 treatment. In the Pilo-matched control group, diazepam treatment alone did not cause iron accumulation and PTGS2 mRNA.

The results of iron measurement indicated that iron accumulation was observed in the hippocampus of PTZ kindling and Pilo-induced seizures (Figures 3A,B). Administration of Fer-1 remarkably reduced hippocampal iron content in PTZ- and Pilo-treated mice (Figures 3A,B). PTGS2 mRNA was decreased in both seizure models after Fer-1 treatment (Figures 3C,D).

### Fer-1 Inhibits Lipid Peroxidation in the Mice Hippocampus of PTZ Kindling and Pilo-Induced Seizures

**FIGURE 4:**
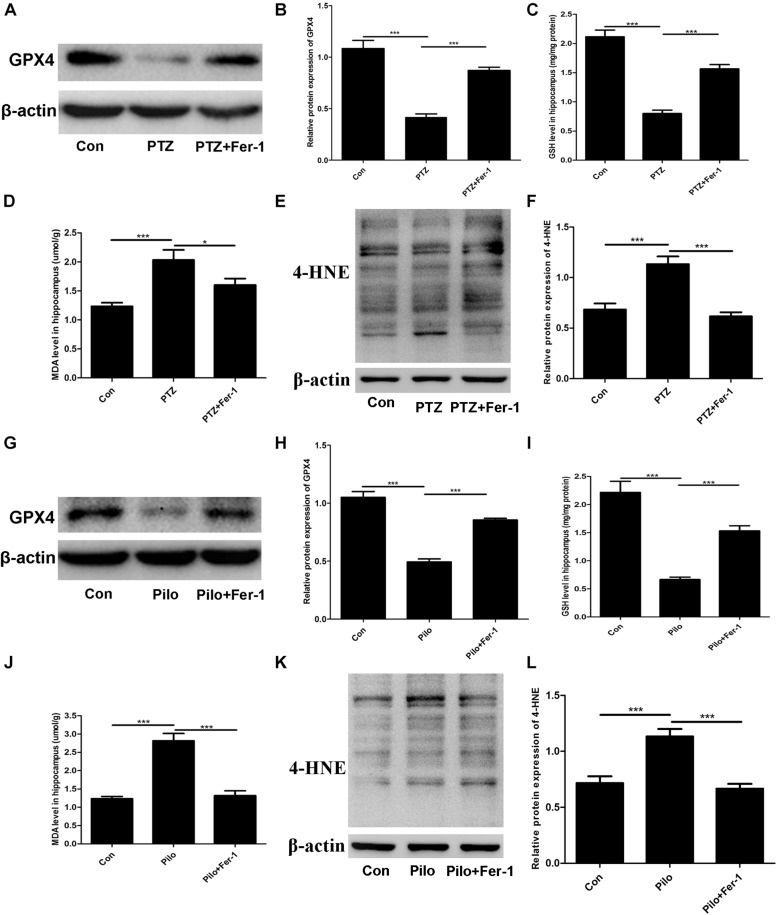
Fer-1 enhances GPX4 protein expression and GSH level as well as decreasing MDA content and the 4-HNE level in the hippocampus of PTZ- and Pilo-treated mice. **(A**,**B**,**G**,**H)** GPX4 protein expression in the hippocampus of PTZ- and Pilo-treated mice (^∗∗∗^*p* < 0.001, *n* = 4). **(C**,**I)** Detection of the GSH level in the hippocampus of PTZ- and Pilo-treated mice (^∗∗∗^*p* < 0.001, *n* = 6). **(D**,**J)** Detection of the MDA content in the hippocampus of PTZ- and Pilo-treated mice (^∗∗∗^*p* < 0.001; ^*^p < 0.05, *n* = 6). **(E**,**F**,**K**,**L)** Detection of the 4-HNE level in the hippocampus of PTZ- and Pilo-treated mice (^∗∗∗^*p* < 0.001, *n* = 6). In the Pilo-matched control group, diazepam treatment alone did not have any effect on the GPX4, GSH, MDA, and 4-HNE levels.

The lipid peroxidation in the hippocampus in our current work was assessed by detecting the GPX4 protein expression, GSH level, MDA content, and 4-HNE level. As shown in Figure 4, enormous lipid peroxidation was found, including decreased GPX4 protein expression, reduced GSH level, increased MDA content, and 4-HNE level in the hippocampus of mice subjected to PTZ kindling and Pilo-induced seizures, and this effect was reversed by Fer-1.

## Discussion

Recurrent and spontaneous seizures is a common phenomenon in the etiology of epilepsy. Most of the traditional drugs exert neuroprotection or anti-epileptic potential by manipulating seizure severity and seizure frequency (Kwan et al., 2011; Fiest et al., 2017), indicating that figuring out the molecular mechanism on how a seizure generates is of vital importance. Hippocampal neuronal loss is a major pathological characteristic of human epilepsy. Multiple cell death modes including apoptosis, necroptosis, autophagy, and pyroptosis have been involved in hippocampal neuronal loss, subsequently aggregating epileptic progress (Cai et al., 2017; Wang J. et al., 2017; Li et al., 2018a; Wu and Wang, 2018). Suppression of these cell death processes can alleviate seizure-induced hippocampal damage and our present investigation provided direct evidence showing the presence of ferroptosis in PTZ kindling and Pilo-induced seizures in mice. More importantly, treatment with ferroptosis inhibitor Fer-1 ameliorated seizure severity and seizure frequency, highlights the potential therapeutic value for curing seizure-associated diseases such as epilepsy by targeting the ferroptosis process.

Pentylenetetrazole kindling and Pilo-induced seizures are ideal models for studying absence epilepsy and status epilepticus, respectively (Duy et al., 2017; Wu and Wang, 2018). These two models have been widely used for studying the process of epileptogenesis and develop novel anti-epileptic drugs. In our current work, PTZ kindling was established through the repeated injection with a sub-convulsive dose of PTZ in mice and almost all mice exhibited consecutive stage 4 seizures by the final dose, which was in line with previous investigations (Zhu et al., 2016). Our current results first revealed the occurrence of ferroptosis in PTZ kindling, thus, future studies are essential to explore the regulatory mechanism of the ferroptosis process in this model. The ramping-up dosing protocol of the Pilo injection was also selected in our present study, as in our preliminary experiments, the high rate of lethality was observed by an injection with the dose of 300 mg/kg Pilo. It was found that three injections with the 100 mg/kg dose are sufficient for the induction of continuous seizure activity, which was consistent with a previous study (Muller et al., 2009).

Ferroptosis is a novel type of RCD and has been reported to be involved in multiple diseases including cancers (Alvarez et al., 2017), neurodegeneration (Hambright et al., 2017), and renal failure (Friedmann Angeli et al., 2014). At least two key factors such as iron and manipulation of lipid peroxidation are indispensable for the execution of ferroptosis (Dixon and Stockwell, 2014). Thus, we hypothesized that brain pathology including PTZ kindling or Pilo-induced seizures is vulnerable to ferroptosis, as the brain contains high-rich phospholipids which are easily subject to lipid peroxidation (Doll et al., 2017). We found that shrunken mitochondria and the upregulation of PTGS2 mRNA, two features of ferroptosis, in PTZ- and Pilo-treated mice, confirmed the presence of ferroptosis in epileptic seizures. A previous study reported the occurrence of ferroptosis in kainic acid-induced epileptic rats, which was similar to our current investigation (Ye et al., 2019). Additionally, in our current work, treatment with the specific ferroptosis inhibitor Fer-1 remarkably alleviated seizures in murine models of PTZ- or Pilo-injection. However, prior work did not observe the ameliorative effect of seizures after treatment with Fer-1 in kainic acid-injected rats. The discrepancy may be attributable to the different methods of seizure model preparation and the use of different species.

As a key regulator of ferroptosis, GPX4 inhibition was previously found to trigger renal failure and exacerbate cognitive deficits via inducing ferroptotic cell death in mice (Friedmann Angeli et al., 2014; Hambright et al., 2017). Evidence for a critical role of GPX4 in epilepsy has arisen from the results showing that GPX4 is a selenium-dependent enzyme for interneuron development and prevention of epileptic seizures (Wirth et al., 2010; Ingold et al., 2018). Our present investigation demonstrated the reduction of GPX4 protein expression in mice treated with PTZ and Pilo. And Fer-1 restored the GPX4 protein level. Consistently, kainic acid-treated rats also exhibited GPX4 reduction and this effect was reversed by Fer-1 (Ye et al., 2019).

In summary, our present work uncovers a novel type of cell death mode, ferroptosis, in PTZ kindling and Pilo-induced seizures and treatment with ferroptosis inhibitor Fer-1 significantly mitigates seizures in PTZ- and Pilo-treated mice.

## Data Availability

The raw data supporting the conclusions of this manuscript will be made available by the authors, without undue reservation, to any qualified researcher.

## Ethics Statement

All animal care and procedures throughout the study were approved by the Ethical Committee of the Animal Centre of Central South University.

## Author Contributions

X-YM and W-LJ designed the study. X-YM wrote the manuscript. W-LJ and H-HZ revised the manuscript.

## Conflict of Interest Statement

The authors declare that the research was conducted in the absence of any commercial or financial relationships that could be construed as a potential conflict of interest.
